# Editorial: Community series in adenosine pathways in cancer immunity and immunotherapy, volume II

**DOI:** 10.3389/fimmu.2025.1538930

**Published:** 2025-01-29

**Authors:** Junjiang Fu, Luca Antonioli, Xiaoli Zheng, Ali H. El-Far

**Affiliations:** ^1^ Key Laboratory of Epigenetics and Oncology, the Research Center for Preclinical Medicine, Southwest Medical University, Luzhou, Sichuan, China; ^2^ Department of Clinical and Experimental Medicine, University of Pisa, Pisa, Italy; ^3^ Department of Immunology, the School of Basic Medical Sciences, Southwest Medical University, Luzhou, China; ^4^ Department of Biochemistry, Faculty of Veterinary Medicine, Damanhour University, Damanhour, Egypt

**Keywords:** adenosine, cancer immunity, cancer immunotherapy, adenosine signaling, immunotherapy, immune checkpoint inhibitor, CAR T cells

## 1 Cancer immunity and immunotherapy

Cancer immunity refers to the impressive ability of the immune system to recognize and eliminate cancerous cells in the body. This complex defense mechanism involves various types of immune cells, including T and natural killer cells. These cells work together to identify abnormal cells that may lead to tumor formation, thereby protecting the body from cancer progression. By effectively distinguishing between healthy cells and those that could be harmful, the immune system plays a vital role in maintaining overall health and preventing the spread of cancer (1). This process is essential because cancer cells can develop from normal cells and may find ways to evade the immune response. Understanding and enhancing cancer immunity is crucial in cancer research and treatment, as these efforts could lead to more effective therapies and better patient outcomes.

Immunotherapy is gaining recognition as a significant approach to treating various types of cancer. This method includes innovative techniques, such as immune checkpoint inhibitors and CAR T-cell therapies, which empower the body’s immune system to combat cancer more effectively. Nevertheless, a key challenge is optimizing these treatments to accommodate a broader range of patients and various tumor types (2). Researchers emphasize the tumor microenvironment—the area surrounding a cancerous tumor that can influence treatment efficacy. A key component of this environment is adenosine signaling. Tumors can manipulate this pathway to deceive the immune system, preventing it from launching an attack. Consequently, targeting adenosine signaling is promising to improve cancer therapies (3, 4).

Immune checkpoint inhibitors block proteins that suppress immune responses, enhancing the immune system’s ability to attack cancer cells. CAR T-cell therapy modifies a patient’s T cells to express chimeric antigen receptors (CARs) that target specific cancer antigens, allowing these cells to identify and destroy cancer effectively. However, tumors often create hostile environments that suppress immune responses, partly through adenosine signaling, which inhibits T-cell activity. Ongoing research aims to understand these interactions and improve immunotherapy effectiveness by targeting pathways like adenosine signaling. This would ultimately enhance the body’s natural immune responses and develop better cancer treatments (5).

## 2 Adenosine and adenosine receptors

**Figure 1:**
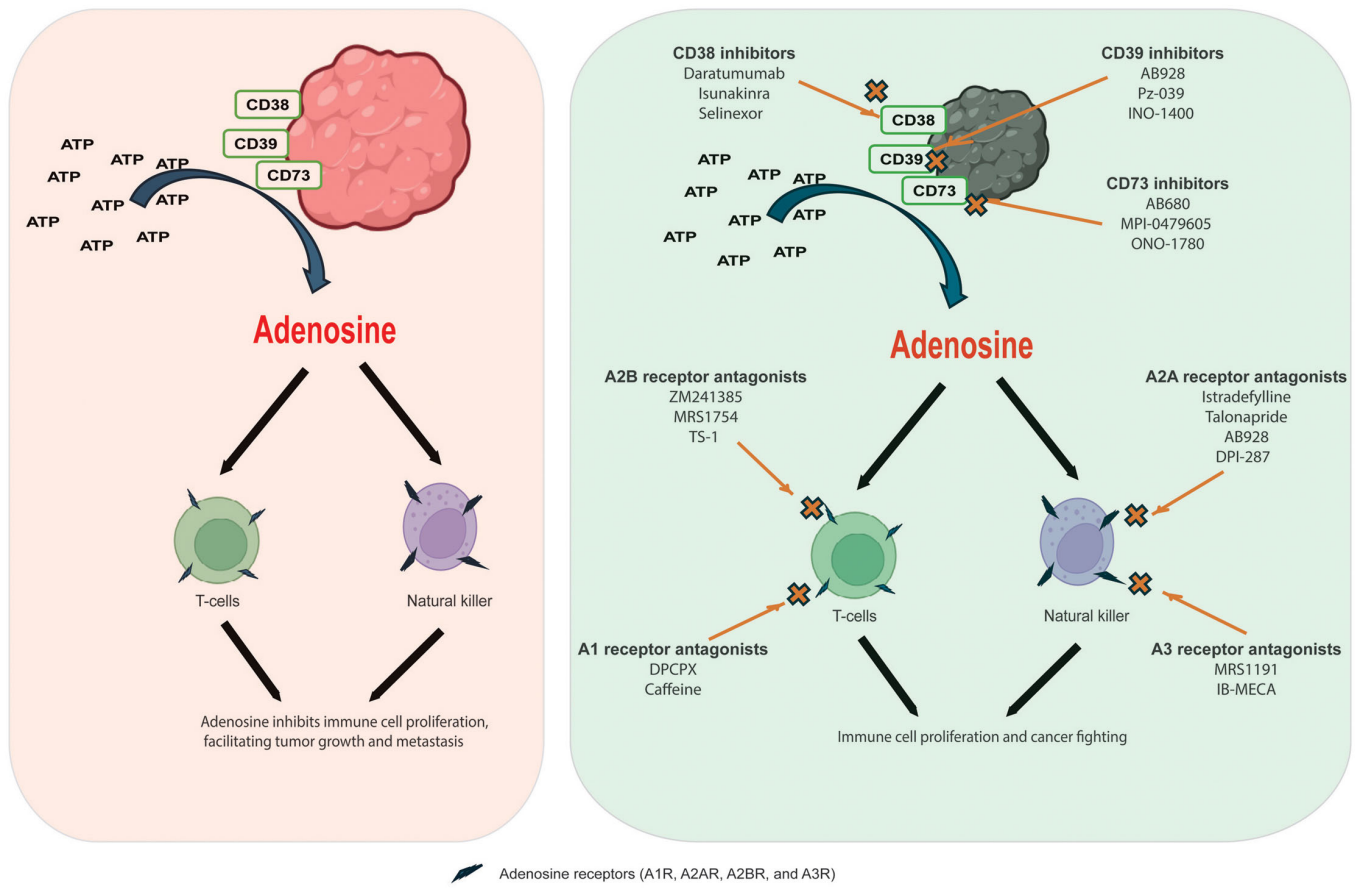
Adenosine pathway targeting for cancer immunotherapy. Left panel: Adenosine generates from ATP by adenosine-generating enzymes, including CD38, CD39, and CD73; after which, adenosine binds to adenosine receptors of natural killer and T-cells inhibiting immune cell proliferation and facilitating tumor growth and metastasis. Right panel: targeting of adenosine pathway with adenosine receptor antagonists and adenosine-generating enzymes inhibitors.

Adenosine, a purine nucleoside, is primarily produced in the body under low oxygen conditions, such as those found in the tumor microenvironment. Elevated adenosine levels act as a potent immunosuppressive metabolite. This compound influences innate and adaptive immune systems by binding specific receptors on immune cells, inhibiting their activation and function. Tumors exploit adenosine signaling to evade the immune response, promoting an altered immune landscape that supports tumor growth and metastasis. Tumor cells generate adenosine through the enzymatic activities of ecto-nucleotide triphosphate diphosphohydrolase-1 and ecto-5’-nucleotidase, which convert ATP to adenosine. This process leads to an altered immune landscape that promotes tumor growth and metastasis. Understanding how adenosine affects our immune system is crucial for developing effective treatments for diseases like cancer. Elevated adenosine levels, primarily produced in hypoxic tumor conditions, act as an immunosuppressive metabolite, inhibiting the activation and function of T cells and natural killer (NK) cells, which are vital for fighting off cancer (6). Adenosine pathway targeting for cancer immunotherapy are summarized on **Figure 1**.

Adenosine binds to receptors on immune cells, suppressing the immune response by inhibiting T cell proliferation, reducing cytokine production, and promoting regulatory T cell differentiation. This creates conditions that allow cancer cells to evade detection, supporting tumor growth and metastasis while hindering an effective immune response. Additionally, it promotes the accumulation of other cells that suppress the immune response (4). Therefore, targeting adenosine pathways could help enhance the body’s natural defenses against tumors and improve the effectiveness of existing therapies (Gardani et al.). In addition, some adenosine derivatives may play roles for targeted gene (7).

Adenosine receptors are G protein-coupled receptors that mediate the effects of adenosine, a purine nucleoside. There are four main types: A1, A2A, A2B, and A3, each with specific functions and tissue distributions. Among them, A1 receptors are primarily inhibitory in function and are predominantly located in the central nervous system and various tissues throughout the body. When these receptors are activated, they initiate a cascade of effects that typically decrease cellular activity. This process also inhibits the release of neurotransmitters essential for communication between nerve cells. Activating A1 receptors can lead to several physiological effects, including a significant reduction in heart rate and enhanced sleep promotion, making them essential players in regulating cardiovascular function and sleep patterns. A2A receptors are excitatory in the brain, immune system, and vascular smooth muscle. They promote vasodilation and regulate immune responses, mainly linked to the immunosuppressive effects of adenosine in tumors. When activated, they inhibit T-cell activation and support tumor growth. Moreover, A2B receptors are present in various tissues, mediating inflammation and allergic responses. They also enhance vascular permeability and promote angiogenesis in tumor biology. Meanwhile, A3 receptors are involved in anti-inflammatory responses and can promote apoptosis in specific cells. They found in tissues like the brain, lungs, and heart and may play roles in asthma and cancer (8).

In cancer, manipulating adenosine signaling through these receptors enables tumors to create an immunosuppressive environment, evading detection and destruction by the immune system. Targeting adenosine receptors, especially A2A and A2B, is being explored as a promising strategy in cancer immunotherapy to enhance the immune response against tumors. The ability to block or antagonize these receptors could help reinvigorate T-cell activity and improve clinical outcomes in cancer patients (4).

## 3 Targeting of adenosine pathway for cancer therapy

Current cancer immunotherapy strategies aim to block adenosine signaling by inhibiting its production and receptors. This enhances the activity of immune cells like T cells and natural killer cells, potentially rejuvenating the immune response against tumors. Combining adenosine receptor antagonists with immune checkpoint inhibitors can enhance antitumor effects beyond single-agent therapies, highlighting the need for personalized treatment plans considering each tumor’s unique microenvironment. Targeting these adenosine pathways presents a promising strategy for enhancing cancer immunotherapy. Researchers aim to restore the immune system’s ability to combat cancer by inhibiting enzymes responsible for adenosine production or blocking adenosine receptors. This can involve combining adenosine receptor antagonists with other immunotherapeutic agents, such as immune checkpoint inhibitors, to achieve synergistic antitumor effects. Current initiatives in cancer immunotherapy are increasingly directing attention toward inhibiting adenosine signaling. Approaches that block adenosine-generating enzymes, such as CD26 and CD39, or antagonize adenosine receptors have the potential to enhance the activity of T cells and natural killer cells (Han et al.). By disrupting the immunosuppressive effects of adenosine, these strategies aim to rejuvenate the immune response against tumors. Targeting of Tim3 and A2a receptors to enhance CAR T cell therapy for solid tumors. However, caution is needed, as single knockdown MSLN-CAR T cells resulted in decreased survival in mice, emphasizing the importance of careful efficacy assessments (Soltantoyeh et al.). Besides, A disintegrin and metalloproteinase domain-10 (ADAM10) is a cell surface protein that cleaved numerous proteins; adenosine inhibited the expression of ADAM10 and modulated the immune in different malignant cancers (Zhang et al.).

### 3.1 Adenosine receptor antagonists

Adenosine receptor antagonists block adenosine’s action at its receptors, counteracting its immunosuppressive effects in the tumor microenvironment. By targeting the A1, A2A, A2B, and A3 receptors, these compounds enhance immune responses against cancer cells. A2A receptor antagonists have been extensively studied as agents in cancer immunotherapy. By blocking A2A receptors, these antagonists can restore T-cell proliferation and boost cytokine production. This enhancement allows the immune system to identify and attack tumor cells more effectively. Istradefylline (KW-6002) is a selective A2A receptor antagonist that has primarily been studied for its effects on Parkinson’s disease. However, ongoing research also investigates its potential applications in cancer treatment. Another selective A2A receptor antagonist, talonapride, has shown promising results in preclinical studies. Additionally, AB928 is a dual antagonist of A2A and A2B receptors tested with immune checkpoint inhibitors in clinical trials. Moreover, DPI-287 is another compound that targets A2A receptors and is currently being investigated for its potential role in cancer therapy.

A2B receptor antagonists specifically target A2B receptors, which have demonstrated beneficial effects across various tumor types. These receptors are instrumental in promoting vascular permeability and angiogenesis—processes that tumors utilize for growth. ZM241385 and MRS1754 are selective A2B receptor antagonists commonly employed in research to investigate the pharmacological effects of inhibiting this receptor. Additionally, TS-1 is a compound that has shown efficacy in blocking the A2B receptor and may be further explored for its therapeutic potential in cancer treatment.

A1 and A3 receptor antagonists are infrequently targeted in cancer therapy. A1 antagonists may lead to an elevated heart rate and an increase in neurotransmitter release, whereas A3 antagonists have the potential to enhance immune responses due to their anti-inflammatory properties. DPCPX (1,3-dipropyl-8-cyclopentylxanthine) is a selective A1 receptor antagonist commonly used in research to study these receptors—additionally, caffeine functions as a non-selective A1 antagonist, promoting the release of neurotransmitters. Furthermore, A3 receptor antagonists such as MRS1191 and IB-MECA have been investigated for their therapeutic effects in different contexts, including cancer.

Several adenosine receptor antagonists are currently under investigation in clinical trials. These compounds promise to improve cancer patients’ outcomes, especially when combined with other therapies such as immune checkpoint inhibitors or CAR T-cell therapy. By inhibiting adenosine signaling, these antagonists can potentially mitigate the immunosuppressive environment created by tumors, thereby revitalizing the body’s natural immune defenses against cancer.

### 3.2 Adenosine-generating enzymes’ inhibitors

Targeting enzymes that produce adenosine, like CD38, CD39, and CD73, is a promising strategy to enhance the immune response. Inhibiting these enzymes reduces adenosine levels, which suppress immune cell activity. Additionally, antagonists that block adenosine receptors can improve T cell and natural killer cell function. This combined approach could significantly boost the body’s ability to combat infections and tumors, making it an exciting avenue for therapy.

Daratumumab is a monoclonal antibody primarily employed in the treatment of multiple myeloma. It binds to CD38 on the surface of myeloma cells, thereby marking them for destruction by the immune system. Another promising CD38 inhibitor, isunakinra, is currently undergoing clinical trials for its potential applications across various cancers, as it targets the immunosuppressive effects of adenosine. Additionally, selinexor has shown some effectiveness in targeting CD38, although its primary mechanism revolves around inhibiting the XPO1 protein.

AB928 serves as a dual antagonist of adenosine receptors by inhibiting the activity of both CD39 and CD73 enzymes. This inhibition effectively disrupts the biochemical pathway responsible for the accumulation of adenosine, a molecule that can suppress the immune response. In contrast, Pz-039 is a targeted small molecule inhibitor targeting CD39, altering adenosine metabolism in the complex tumor microenvironment and potentially enhancing anti-tumor immunity. Additionally, INO-1400 is an innovative investigational product designed to inhibit CD39, and it is currently under study to explore its promising applications in immunotherapy to harness the body’s immune system to combat cancer more effectively (9).

CD73 inhibitors include AB680, a selective small molecule that has shown promise in preclinical studies and is currently being evaluated in clinical trials for its ability to enhance anti-tumor immune responses. Another selective CD73 inhibitor, MPI-0479605, targets the immunosuppressive tumor microenvironment, mainly when used alongside other cancer therapies. Additionally, eflornithine has been recognized for its potential impact on CD73 activity in specific contexts. Moreover, ONO-1780 is a novel small-molecule inhibitor that targets CD73 and is presently undergoing clinical evaluation to assess its effectiveness against various malignancies (10).

Researchers are currently exploring inhibitors of adenosine-generating enzymes due to their promising potential to enhance immune function. By targeting and decreasing the production of adenosine within the tumor microenvironment, these inhibitors may help mitigate the immunosuppressive effects often linked to tumors. This strategy could ultimately play a crucial role in improving cancer treatment outcomes.

## 4 Conclusion

A growing body of preclinical research has highlighted the exciting potential of therapies that target adenosine. One noteworthy example is the combination of adenosine receptor antagonists with immune checkpoint inhibitors. This powerful combination has produced synergistic effects, resulting in a significant increase in antitumor activity compared to treatments using a single agent. These findings underscore the critical importance of carefully designed combination strategies, especially in the context of personalized treatment regimens. Such regimens consider the unique characteristics of each patient’s tumor microenvironment, tailoring approaches to optimize therapeutic outcomes and enhance patient care.

To fully utilize the therapeutic potential of adenosine-targeted treatments, ongoing research is essential to explore the complex mechanisms behind the production and signaling of adenosine in the tumor microenvironment. This environment is marked by a unique interaction among various cellular components and biochemical signals, which requires a deeper understanding of how adenosine is generated and used in tumor biology. Researchers can confirm their importance in tumorigenesis by identifying and investigating new therapeutic targets within the adenosine pathway. Such studies are crucial for unraveling the complexities of cancer development and are vital for creating innovative and effective strategies for treating malignancies.

## References

[1] Aktar N Yueting C Abbas M Zafar H Paiva-Santos AC Zhang Q. Understanding of immune escape mechanisms and advances in cancer immunotherapy. J Oncol. (2022) 2022:8901326. doi: 10.1155/2022/8901326 35401745 PMC8989557

[2] Fu J Antonioli L El-Far AH. Editorial: Adenosine pathways in cancer immunity and immunotherapy. Front Immunol. (2023) 14:1298487. doi: 10.3389/fimmu.2023.1298487 37885877 PMC10598335

[3] Yang H Zhang Z Zhao K Zhang Y Yin X Zhu G. Targeting the adenosine signaling pathway in macrophages for cancer immunotherapy. Hum Immunol. (2024) 85:110774. doi: 10.1016/j.humimm.2024.110774 38521664

[4] Vijayan D Young A Teng MWL Smyth MJ. Targeting immunosuppressive adenosine in cancer. Nat Rev Cancer. (2017) 17:765. doi: 10.1038/nrc.2017.110 29162946

[5] de Visser KE Joyce JA. The evolving tumor microenvironment: From cancer initiation to metastatic outgrowth. Cancer Cell. (2023) 41:374–403. doi: 10.1016/j.ccell.2023.02.016 36917948

[6] Hasko G Cronstein BN. Adenosine: an endogenous regulator of innate immunity. Trends Immunol. (2004) 25:33–9. doi: 10.1016/j.it.2003.11.003 14698282

[7] Du J Fu J Zhang W Zhang L Chen H Cheng J. Effect of DPP4/CD26 expression on SARS−CoV−2 susceptibility, immune response, adenosine (derivatives m(6)(2)A and CD) regulations on patients with cancer and healthy individuals. Int J Oncol. (2023) 62:41. doi: 10.3892/ijo.2023.5489 36799191 PMC9946808

[8] Mazziotta C Rotondo JC Lanzillotti C Campione G Martini F Tognon M. Cancer biology and molecular genetics of A(3) adenosine receptor. Oncogene. (2022) 41:301–8. doi: 10.1038/s41388-021-02090-z PMC8755539 34750517

[9] Moesta AK Li XY Smyth MJ. Targeting CD39 in cancer. Nat Rev Immunol. (2020) 20:739–55. doi: 10.1038/s41577-020-0376-4 32728220

[10] Xia C Yin S To KKW Fu L. CD39/CD73/A2AR pathway and cancer immunotherapy. Mol Cancer. (2023) 22:44. doi: 10.1186/s12943-023-01733-x 36859386 PMC9979453

